# Anti-COVID Drugs (MMV COVID Box) as Leishmanicidal Agents: Unveiling New Therapeutic Horizons

**DOI:** 10.3390/ph17030266

**Published:** 2024-02-20

**Authors:** Atteneri López-Arencibia, Carlos J. Bethencourt-Estrella, Desirée San Nicolás-Hernández, Jacob Lorenzo-Morales, José E. Piñero

**Affiliations:** 1 Instituto Universitario de Enfermedades Tropicales y Salud Pública de Canarias, Universidad de La Laguna, Avda. Astrofísico Fco. Sánchez, S/N, 38203 San Cristóbal de La Laguna, Spain; cbethene@ull.edu.es (C.J.B.-E.); dsannico@ull.edu.es (D.S.N.-H.); jmlorenz@ull.edu.es (J.L.-M.); jpinero@ull.edu.es (J.E.P.); 2 Departamento de Obstetricia y Ginecología, Pediatría, Medicina Preventiva y Salud Pública, Toxicología, Medicina Legal y Forense y Parasitología, Universidad de La Laguna, 38203 San Cristóbal de La Laguna, Spain; 3 Centro de Investigación Biomédica en Red de Enfermedades Infecciosas (CIBERINFEC), Instituto de Salud Carlos III, 28220 Madrid, Spain

**Keywords:** treatment, anti-COVID, *Leishmania*

## Abstract

Leishmaniasis, a neglected tropical disease, poses a significant global health challenge, necessitating the urgent development of innovative therapies. In this study, we aimed to identify compounds from the COVID Box with potential efficacy against two *Leishmania* species, laying the foundation for future chemical development. Four promising molecules were discovered, demonstrating notable inhibitory effects against *L. amazonensis* and *L. donovani*. Our study revealed that bortezomib, almitrine, and terconazole induced a significant decrease in mitochondrial membrane potential, while the above compounds and ABT239 induced plasma permeability alterations, chromatin condensation, and reactive oxygen species accumulation, indicating early apoptosis in *Leishmania amazonensis* promastigotes, preventing inflammatory responses and tissue damage, thereby improving patient outcomes. Furthermore, ADME predictions revealed favorable pharmacokinetic profiles for all compounds, with bortezomib and ABT239 standing out as potential candidates. These compounds exhibited intestinal absorption, blood–brain barrier penetration (excluding bortezomib), and good drug-likeness for bortezomib and ABT239. Toxicity predictions for CYP-inhibition enzymes favored bortezomib as the safest candidate. In conclusion, our study identifies bortezomib as a promising aspirant for leishmaniasis treatment, demonstrating potent antiparasitic activity, favorable pharmacokinetics, and low toxicity. These findings emphasize the potential repurposing of existing drugs for neglected diseases and highlight the importance of the COVID Box in drug discovery against tropical diseases.

## 1. Introduction

Protozoa from the *Leishmania* genus are responsible for causing leishmaniasis, a complex parasitic disease prevalent in tropical and subtropical regions. Recognized as a significant global health concern by the WHO, leishmaniasis ranks fourth in morbidity and second in mortality rates among tropical diseases [1,2]. Factors such as poverty, malnutrition, inadequate housing, and limited access to healthcare contribute to its persistence, making it a disease that disproportionately affects the most vulnerable populations. This disease manifests in three primary clinical forms—cutaneous, mucocutaneous, and visceral—depending on the species and the host’s immune response, each with its distinct symptoms and severity [3].

**Figure 1:**
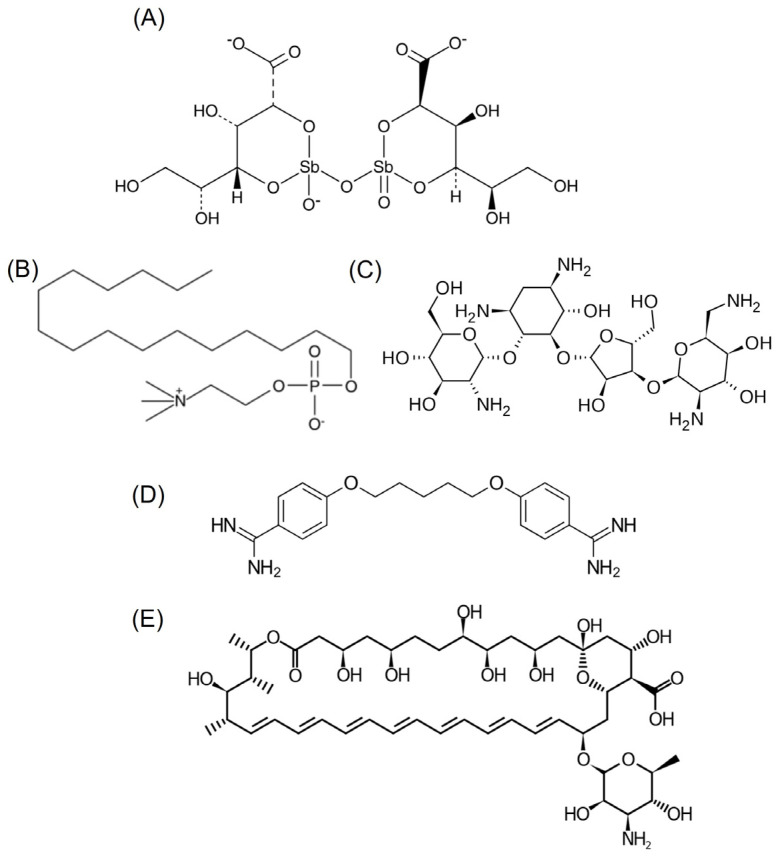
Chemical structure of (**A**) stibogluconate, (**B**) miltefosine, (**C**) paromomycin, (**D**) pentamidine, and (**E**) amphotericin B.

Primary treatments against leishmaniasis involve pentavalent antimonial formulations and sodium stibogluconate, although alternatives like miltefosine, amphotericin B, paromomycin, and pentamidine exist (Figure 1). Furthermore, treatment options for leishmaniasis face hurdles. The existing medications often come with drawbacks such as high costs, toxicity, and challenges in administration. The emergence of drug-resistant strains adds another layer of complexity, rendering some conventional treatments less effective and posing a serious threat to disease management [4,5]. The issue of treatment failure is significant, influenced by various factors, including host immunity, parasite drug resistance, and environmental changes like the spread of the disease to new areas due to global warming. The emergence of resistant strains compounds this problem, as studies increasingly show resistance to current treatments, posing a new challenge for the WHO in developing the next generation of leishmaniasis treatment [6]. In response to these challenges, there is growing interest in exploring alternative approaches to tackle leishmaniasis. One such avenue involves the repurposing of already known drugs, harnessing their potential against this parasitic infection. Repurposing existing drugs offers a promising strategy, leveraging established safety profiles, known pharmacokinetics, and prior clinical data to expedite the development of novel treatment options for leishmaniasis [7].

Drug repurposing, also termed drug repositioning, holds multiple advantages. Firstly, it significantly saves time and reduces costs compared to the lengthy and expensive process of developing a new drug from scratch, which can take over a decade and billions of dollars. Secondly, repurposed drugs leverage their known safety profiles, having undergone extensive human testing for safety and efficacy, thereby reducing the risk of unexpected adverse effects when used for new purposes. Thirdly, they offer potential in combination therapies, complementing existing treatments to enhance effectiveness or combat resistance. Moreover, drug repurposing serves as a viable strategy for addressing rare and neglected diseases, filling the void left by limited commercial incentives for new drug development. Lastly, this approach can unveil fresh insights into disease mechanisms or novel actions of drugs. While promising in expediting drug discovery, it is imperative to underscore the necessity of rigorous scientific research and clinical trials to validate the efficacy of repurposed drugs for new indications.

The advantages of drug repurposing, coupled with the priority given by researchers to the development of innovative, safe, and effective anti-leishmanial molecules with minimal side effects, have led the scientific community to actively search for known compounds with leishmanicidal properties. The COVID Box library of Medicines for Malaria Venture (MMV, Switzerland) generously provides 160 diverse compounds with activity against SARS-CoV-2 (COVID-19). These compounds exhibit diverse bioactivities, and each of them shows suspected activity or potential against SARS-CoV-2, the virus responsible for the current pandemic. Surprisingly, these compounds have shown promise not only against the new coronavirus but also against different infectious agents, such as protozoan parasites [8]. This study focuses on screening and studying these molecules for MMV COVID Box, with the aim of contributing to the search for better treatment options.

When making decisions about antiparasitic drugs, it is important to consider the type of death the compound will produce in the parasite. Killing a parasite through necrosis is a messy and uncontrolled process that can cause inflammation in the surrounding tissue due to the release of a variety of substances, including toxins and antigens, into the surrounding tissue. This can trigger a strong immune response, leading to inflammation and tissue damage. In severe cases, this inflammation can cause significant tissue damage and may lead to organ dysfunction. Therefore, while killing the parasite is necessary, it is important that it is conducted in a controlled manner to minimize these potential negative effects. In contrast, apoptotic death is the best in terms of the host immune system’s reaction. In observing the programmed cell death (PCD) or apoptosis-like process in the parasite, numerous morphological changes become apparent, including cytoplasm condensation, reduced cell volume, diminished mitochondrial membrane potential, chromatin condensation, and DNA fragmentation [9,10]. In apoptotic cells, the plasma membrane undergoes phospholipid asymmetry loss, leading to the externalization of phosphatidylserine (PS), which becomes exposed to the extracellular environment, facilitating recognition via phagocytic cells like macrophages [11].

This article will provide a comprehensive overview of the rationale behind drug repurposing, elucidating the advantages and challenges associated with this approach. Additionally, it will highlight the methodologies employed in screening and evaluating the efficacy of known drugs against *Leishmania* parasites, offering insights into the potential mechanisms of action and their relevance in combating this debilitating disease. We aim to contribute to the growing body of knowledge in the quest for innovative and effective treatments against leishmaniasis. The findings from this work endeavor hold the potential to bridge the gap in treatment options, offering renewed hope in the fight against this neglected tropical disease.

## 2. Results

### 2.1. Screening of the Box

**Figure 2:**
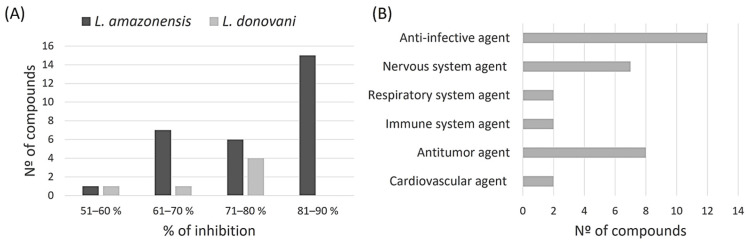
The initial screening results enclosed 160 molecules. The data specifically illustrate molecules that cause a parasite inhibition exceeding 51% at 10 μM. (**A**) categorizes molecules based on their inhibition percentage against *L. amazonensis* and *L. donovani* and (**B**) classifies the active molecules according to their biological activity.

After initial screening of all compounds present in the COVID Box, we identified some that demonstrated leishmanicidal activity against *L. amazonensis* and *L. donovani* promastigotes, showing growth inhibition equal to or greater than 51% when these compounds were assayed at 10 μM (Figure 2). Of the 160 compounds, 32 met this criterion for *L. amazonensis*, with the majority showing growth inhibition ranging from 81–90% (15 compounds). For *L. donovani*, of the 160 compounds tested, only 6 met this inhibition criterion, and most showed inhibitions ranging from 71 to 80% (4 compounds).

Moreover, the majority of these compounds are categorized as anti-infective agents, with 12 compounds (5 antimalarial and 4 antifungals within others), followed by antitumor agents with 8 compounds (leukemia, myeloma, and lymphoma), and nervous system agents with 7 compounds (antidepressant and antipsychotic).

To determine which compounds were more selective against the parasite, another screening of the compounds, also at 10 μM, was carried out, this time against murine macrophages, to test their cytotoxicity. The following graph shows a scatter plot representing each compound with its activity and toxicity. The points located in the lower right part will represent the compounds that show higher antiparasitic activity with lower cytotoxicity against macrophages.

**Figure 3:**
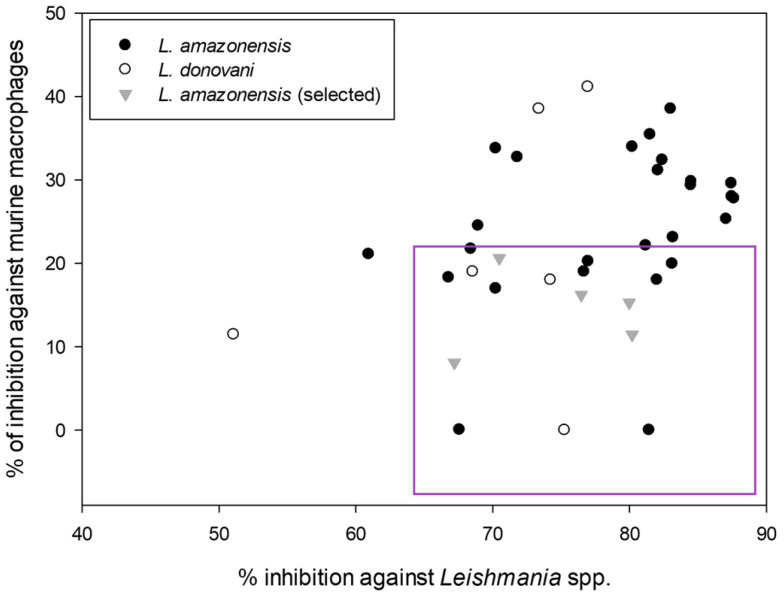
Screening of the leishmanicidal activity against the promastigote stage of the best compounds from MMV COVID Box and their cytotoxic activity against murine macrophages. All expressed as % of inhibition at 10 μM.

After obtaining the compounds with the best selectivity profile against parasites (lower IC_50_ and higher CC_50_), a literature search was performed to discard compounds already studied on parasites, and after this step, products not available on the market for purchase were excluded. Bortezomib, terconazole, almitrine, midostaurin, and ABT239 were the molecules selected for further studies (represented as gray triangles in Figure 3).

### 2.2. Leishmanicidal and Cytotoxic Activity

Subsequently, we assessed the inhibitory effects of these five molecules on both promastigote strains forms of *Leishmania amazonensis* and *L. donovani*, detailed in Table 1. Among the results, Bortezomib and Midostaurin exhibited the highest activity against *L. amazonensis*, displaying IC_50_ values of 0.42 and 0.67 μM, respectively, followed by Almitrine and ABT239, each with IC_50_ values below 1.4 μM. The molecular structures of the five compounds are depicted in Table 2. In contrast, the compound that exhibited the highest activity against *L. donovani* was ABT239, with an IC_50_ value of 1.68 μM, followed by Terconazole and Bortezomib, displaying IC_50_ values of 11.61 and 37.20 μM, respectively.

Given that the strain *L. amazonensis* exhibited the most significant biological activity, it was chosen for further studies. The subsequent step involved assessing the biological activity against the intracellular form of the amastigotes. Table 3 presents the IC_50_ values for the compounds that demonstrated the highest activity against amastigotes, along with the selectivity index derived from comparing the leishmanicidal activity values with the cytotoxicity values.

In this scenario, bortezomib and ABT239 displayed the most potent biological activities against the intracellular stage of the parasite, with IC_50_ values of 0.04 and 0.49 µM, respectively. This was followed by almitrine and terconazole, both of which had IC_50_ values below 2.4 µM. The selectivity indices observed vary between compounds due to their cytotoxicity, but it is important to highlight that three of the compounds exhibit SI values better than miltefosine, with bortezomib standing out with an SI with a value of 2500. Interestingly, midostaurin, which had been very active against the promastigote stage of the parasite, showed no activity against the amastigote form of the parasite at 10 μM, so it was discarded from future studies.

### 2.3. Study of Cell Death

Given that four compounds demonstrated a potent leishmanicidal activity and exhibited moderate cytotoxicity, all were selected for additional investigations to determine the nature of cell death they induce in the parasite.

**Figure 4:**
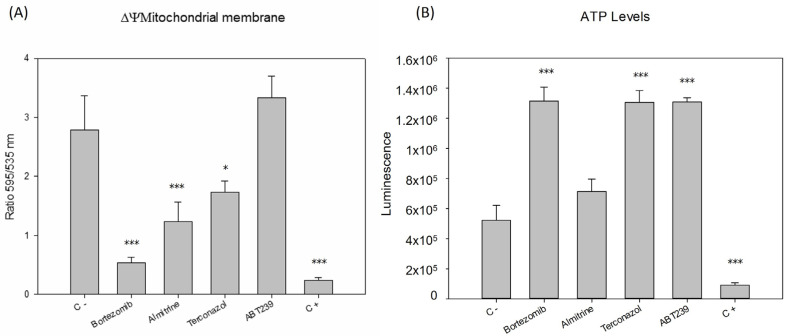
Changes in the (**A**) mitochondrial membrane potential (ΔΨm) and (**B**) ATP levels of *Leishmania amazonensis* promastigotes after 24 h of incubation with the IC_90_ of the compounds. Error bars represent the standard deviations (SD). Each data point indicates the mean of the results of three measurements, (*) *p* < 0.05, (***) *p* < 0.001. C -: untreated parasites. C +: parasites treated with sodium azide.

The mitochondrion, important in this parasite for storing the genetic material known as the kinetoplast, is their singular and unique mitochondria. The initial decision was to examine the impact of the compounds on this crucial organ of the parasite, the organelle that provides it with the energy necessary for its metabolism. Figure 4 illustrates that all compounds, except for ABT239, managed to significantly reduce the membrane potential to levels never lower than the positive control (sodium azide), displaying notable differences compared to the negative control. Subsequently, ATP levels were analyzed to check if they were being modified. It was found that all compounds significantly increased ATP levels compared to the control group of untreated parasites. The level of ATP in a cell can be increased through various metabolic processes, even if the mitochondrial membrane potential is altered. When the ability of parasites to generate ATP through mitochondrial oxidative phosphorylation is compromised, parasites can adapt alternative metabolic pathways, such as increasing their glycolytic activity or the beta-oxidation of fatty acids to maintain their energy supply [12], which may explain the results obtained.

**Figure 5:**
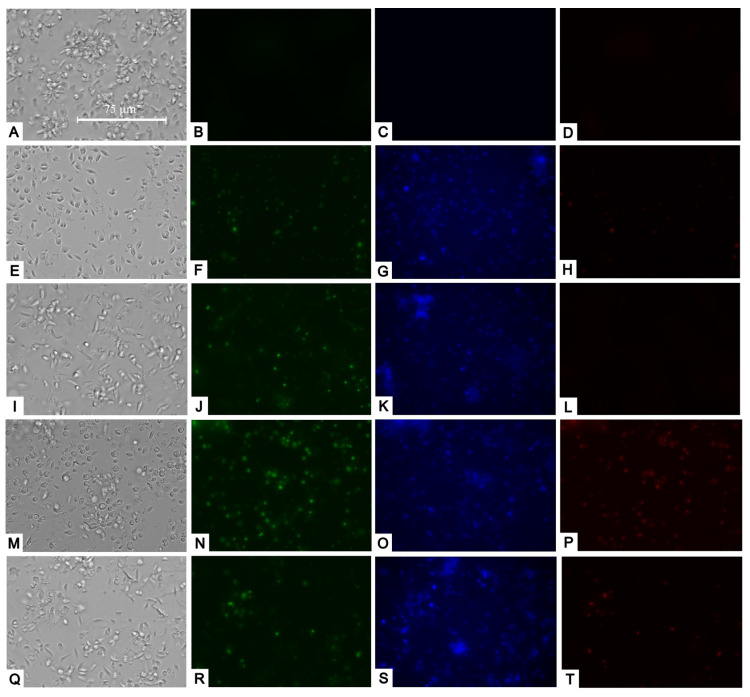
Transmitted light, SytoxGreen (green), Hoechst (blue), and Propidium iodide (red) staining. Results after 24 h of incubation of *L. amazonensis* promastigotes with the IC90 of compounds. Negative control (**A**–**D**). Bortezomib (**E**–**H**); almitrine (**I**–**L**); terconazole (**M**–**P**); and ABT239 (**Q**–**T**). Images were captured using an EVOS FL Cell Imaging system (Thermo Fisher Scientific) (40×). Scale: 75 µm.

Following our assessment of mitochondrial damage, we moved on to examine potential changes in the plasma membrane and its permeability. Utilizing the SytoxGreen reagent, we noticed that the studied compounds caused a slight increase in plasma membrane permeability, as some of the fluorophores managed to infiltrate the parasites and attach to their DNA. This resulted in fluorescence within their nucleus, likely in the most damaged parasites (Figure 5).

Upon examining the staining with Hoechst and propidium iodide, we observed that some parasites exhibited strong blue fluorescence (Hoechst) within their nuclei, suggesting chromatin condensation. In addition, the treatments caused some parasites to be marked in red (propidium iodide), which indicated that they had already died, suggesting that apoptosis was occurring early (Figure 5).

**Figure 6:**
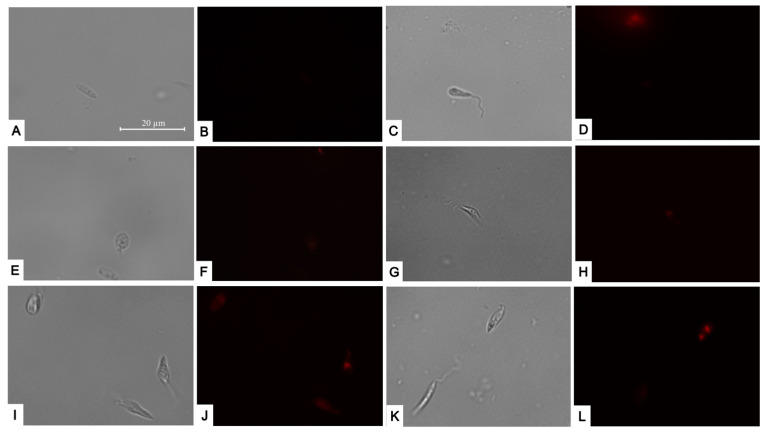
CellROX Deep Red staining. Results after 24 h of incubation of *L. amazonensis* promastigotes with the IC_90_ of the compounds. Negative control (**A**,**B**); Positive control treated with H_2_O_2_ (**E**,**F**); Bortezomib (**C**,**D**); almitrine (**G**,**H**); terconazole (**I**,**J**); and ABT-239 (**K**,**L**). Images were captured using an EVOS FL Cell Imaging system (Thermo Fisher Scientific) (100×). Scale: 20µm.

Given that the most significant and pronounced changes were observed at the mitochondrial level, we decided to investigate the potential effects of reactive oxygen species accumulation within the cells post-treatment. We noticed that the four compounds could trigger a substantial rise in red fluorescence, signifying an increase in the accumulation of reactive oxygen species to levels similar to the positive control with hydrogen peroxide (Figure 6).

### 2.4. ADME-Tox Predictions

**Figure 7:**
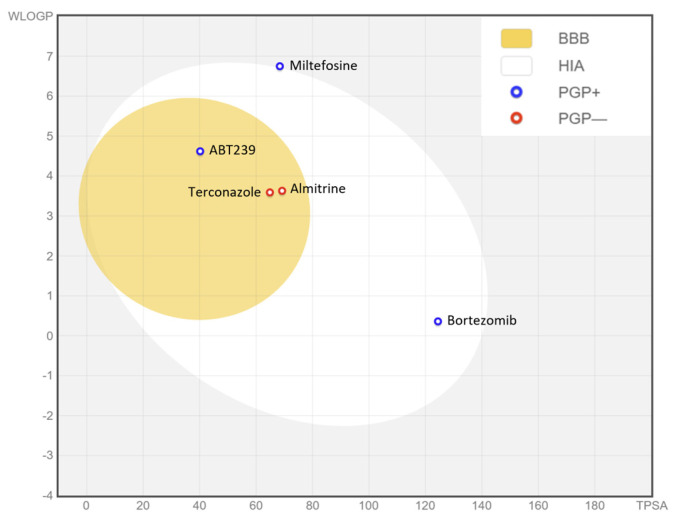
BOILED-Egg graph representing in silico prediction of human intestinal absorption (HIA) and blood–brain barrier (BBB) penetration. The white region is for a high probability of passive absorption by the gastrointestinal tract, and the yellow region is for a high probability of brain penetration. In addition, the points are colored in blue if predicted as actively effluxed by P-gp (PGP+) and in red if predicted as non-substrate of P-gp (PGP−).

When analyzing the ADME-Tox properties of a drug, particularly for leishmaniasis, one of the most desirable factors would be the possibility of oral administration. Thus, we can start this study by assessing gastrointestinal absorption and brain accessibility, crucial pharmacokinetic behaviors throughout the different stages of drug discovery. The Brain Or IntestinaL EstimateD (BOILED-Egg) permeation method is an accurate predictive model that estimates the lipophilicity and polarity of small molecules, ranging from early screening of chemical libraries in drug discovery to the evaluation of potential drug candidates for further development. This method simultaneously predicts permeability in both the brain and gut using the same physicochemical descriptors. Therefore, this prediction was performed for the selected compounds and miltefosine using the swissADME tool, as depicted in Figure 7.

As depicted in the egg graph, all four compounds, as well as miltefosine, are predicted to be drugs that would be well absorbed at the intestinal level, and ABT239, terconazole, and almitrine are also predicted to be able to penetrate the BBB. In addition, ABT239, bortezomib, and miltefosine are predicted to be non-substrates of P-glycoprotein (P-gp), unlike terconazole and almitrine, which would be substrates of this transmembrane pump, and therefore susceptible to being expelled from the cell through the action of P-gp. These P-gp-related predictions are based on a classification-based model using machine learning [13]. P-gp plays a crucial role in the absorption and distribution of drugs in the body, pumping drugs from the inside to the outside of the cell. Therefore, a drug that does not bind to P-gp may have several advantages: increased bioavailability, as P-gp in the gut pumps drugs back into the lumen, decreasing their absorption, or even reduced drug–drug interactions as if a drug does not bind to P-gp, it is less likely to have interactions with drugs that do [14].

The forecast of drug bioavailability is vital for understanding how a drug behaves pharmacokinetically, and it is relevant data in the different stages of drug discovery. The Bioavailability Radar, also performed for each compound, is a rapid evaluation of six physicochemical properties (lipophilicity, size, polarity, solubility, flexibility, and saturation) (see Supplementary Information). The selected compounds generally have their physicochemical parameters within the optimal ranges. ABT239 is the compound that best fits the ideal parameters, followed by almitrine and terconazole, which slightly exceed their size and lipophilicity values. Next would be bortezomib, with excess flexibility in the molecule, a fact that is also observed in the terconazole. Miltefosine stands out with a clear excess of flexibility and lipophilicity in its physicochemical properties.

**Figure 8:**
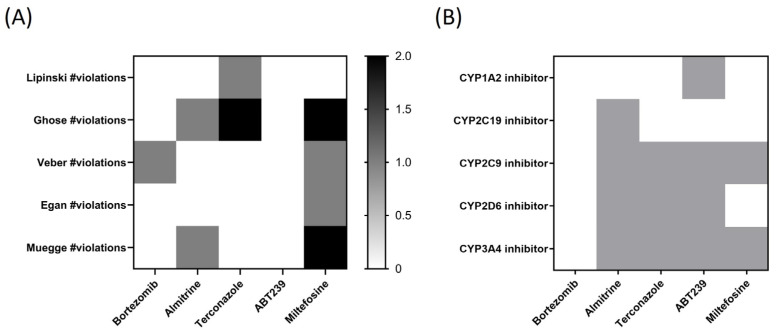
Heatmaps of (**A**) predicted drug-likeness, established as qualitatively the chance for a molecule to become an oral drug with respect to bioavailability, and (**B**) predicted cytochrome P450 (CYP) inhibition. White: no inhibition; Grey: inhibition.

SwissADME also provides access to five distinct rule-based filters that define molecules as oral drug-like, based on various property ranges. These filters stem from analyses conducted by leading pharmaceutical companies seeking to enhance the quality of their exclusive chemical libraries. The Lipinski (Pfizer) filter was the first rule-of-five method introduced [lipophilicity (LogP) < 5, Hydrogen Bond Donor (HBD) ≤ 5, Hydrogen Bond Acceptor (HBA) ≤ 10, molecular weight (g/mol) ≤ 500 g/mol, and flexibility (rotatable bonds) < 10], as well as the Ghose (Amgen Inc.) (−0.4 < LogP < 5.6; HBD ≤ 5 and HBA ≤ 10; MW < 500 g/mol), Veber (GlaxoSmithKline) (flexibility (rotatable bonds): <10; HBD ≤ 5; HBA ≤ 10), Egan (Pharmacia Inc.) (−0.4 < LogP < 5.6; MW < 500 g/mol; HBA ≤ 10)), and Muegge (Bayer AG) (HBD ≤ 5; MW < 500 g/mol) methods, which were adopted by each pharmaceutical company [15]. The values obtained from violations of the rules for the different compounds have been plotted in Figure 8A.

As observed in Figure 8A, ABT239 is the only compound that does not present any violation in the five different rules or filters established by pharmaceutical companies for the qualitative prediction of drug-likeness for orally administered drugs. In the second position would be bortezomib, which only presents one violation of Veber’s rule. Next would be almitrine with two violations (one in Ghose filter and another in Muegge’s rule), and terconazole, with two violations in the Ghose and one in Lipinski’s. Interestingly, the reference drug miltefosine stands out, presenting violations in four of the studied rules (two in Ghose and Muegge and one in Veber and Egan).

A general concern for drug discovery, in terms of toxicity, is avoiding the inhibition of drug-metabolizing cytochromes P450 (CYP). CYP inhibition can lead to decreased drug/chemical elimination, which is a major cause of drug–drug interactions (DDI), provoking adverse events. Therefore, identifying the potential inhibition of CYP is critical for drug development and clinical drug treatment [16]. The five most important human CYP isoforms, regarding drug metabolism, are 1A2, 2C9, 2C19, 2D6, and 3A4, which are involved in ∼95% of the CYP-mediated metabolism of drugs, representing ∼75% of total drug metabolism. In Figure 8B, bortezomib stands out as the compound that does not inhibit any of the cytochromes studied in silico, followed by terconazole, which inhibits three of them, and almitrine and ABT239, which inhibits four of the five studied CYP.

## 3. Discussion

The acute need for innovative therapies for leishmaniasis highlights the persistent challenges posed by this neglected disease. With limited attention from pharmaceutical companies, coupled with restricted research and dissemination, the development of effective treatments for leishmaniasis remains a necessity. In this research context, our primary objective was to identify compounds from the COVID Box capable of effectively combating two different *Leishmania* species and potentially laying the groundwork for future chemical development. This approach led to the discovery of four promising molecules: bortezomib, almitrine, terconazole, and ABT-239. These active compounds demonstrated promising inhibitory effects against the two strains of *Leishmania* studied, exhibiting in vitro IC_50_ values against *L. amazonensis* of 0.42 ± 0.08 µM in promastigote viability upon exposure to bortezomib, an IC_50_ of 1.19 ± 0.02 µM for almitrine, an IC_50_ of 3.82 ± 1.04 µM for terconazole, and an IC_50_ of 1.31 ± 0.07 µM for ABT-239. In the case of *L. donovani*, three active compounds were identified: bortezomib, terconazole, and ABT239, with IC_50_ ranges between 1.68 and 37.2 µM. Additionally, the effect of these compounds against the amastigote stage of the parasite was demonstrated, with IC_50_ values from 0.04 µM for bortezomib to 2.38 µM for terconazole, referring to *L. amazonensis*. This, combined with a cytotoxic concentration 50 ranging from 45 to more than 100 µM among the compounds, results in all of them achieving a selectivity index higher than that obtained for the reference drug miltefosine (except by terconazole), highlighting the selectivity of bortezomib, which is more than 100 times greater than miltefosine.

Effective compounds for the treatment of leishmaniasis must cause controlled parasite elimination by inducing programmed cell death (PCD) in the parasite. Distinctive markers of PCD in the genus *Leishmania* include chromatin condensation, alterations in plasma membrane permeability, increased ROS production and reduced mitochondrial membrane potential [9,10]. In contrast, unregulated cell death lacking typical apoptotic or autophagic features would incite inflammatory responses, worsening disease progression and accelerating the deterioration of the patient’s infected tissue due to the damage of vital parasite-infected tissues (mainly skin, liver, and spleen) by the cytokine storm and cellular debris caused by necrosis. The results of our study showed a significant decrease in the mitochondrial membrane potential of parasites treated with bortezomib, almitrine, and terconazole (not for ABT239), a marked increase in plasma permeability of promastigotes treated with the four selected compounds, as well as an increase in chromatin condensation, often coupled with premature parasite death, indicating early apoptosis. In addition, the selected compounds induced an increase in the accumulation of reactive oxygen species inside the promastigotes.

Furthermore, considering the broad-spectrum activity of the compounds evaluated, they are likely to show significant efficacy against other pathogenic microorganisms. For instance, bortezomib corresponds to a pioneering proteasome inhibitor approved in 2003 by the FDA for the treatment of refractory or relapsed multiple myeloma [17]. It selectively and reversibly disrupts the ubiquitin-proteasome pathway, whose inhibition in the *Leishmania* genus is known to block parasite proliferation and differentiation, thereby altering its virulence factor [18]. On the other hand, terconazole is recognized for its antifungal properties and has exhibited notable activity against *Candida* species [19], as well as against the protozoa *Trypanosoma cruzi* [20]. This azole group belonging to the molecule acts by inhibiting the 14-alpha-demethylase, a key enzyme for ergosterol biosynthesis that has been demonstrated as an essential lipid for the *Leishmania* genus survival [21]. In fact, compounds that bind to ergosterol or inhibit its synthesis, like amphotericin B, itraconazole, ketoconazole, and others, are already used to treat leishmaniasis [22]. Moreover, ABT-239 is a potent H3 histamine receptor antagonist, presenting stimulant and nootropic effects, and has been investigated as a treatment for attention deficit hyperactivity disorder, Alzheimer’s disease, and schizophrenia [23]. Furthermore, almitrine is a diphenylmethylpiperazine derivative classified as a respiratory stimulant used in case of hypoxemia due to obstructive bronchitis. It enhances respiration by acting as an agonist of peripheral chemoreceptors located on the carotid bodies. This means it stimulates these receptors, which in turn enhances the respiratory process [24].

Few researchers have yielded promising findings in prior investigations utilizing the MMV COVID Box, examining its biological activity against parasites. One example is the study by Dos Santos and colleagues, who tested the Box against *Toxoplasma gondii*, finding 29 active compounds against the tachyzoites of the parasite. After in silico predictions of physicochemical properties and drug-likeness, they decided that the best compound to continue their studies was almitrine [25], which was also selected in the present work, highlighting its antiprotozoal activity.

In making ADME predictions for the compounds, we found that all four are predicted to be absorbed at the intestinal level, with all but bortezomib also predicted to be BBB penetrators. Additionally, it should be noted that almitrine is the only compound among those studied that is already administered orally [26]. In addition, bortezomib and ABT239 appear not to be P-gp substrates, potentially increasing their bioavailability and reducing their interaction with other drugs. In addition, all compounds showed a good pharmacokinetic bioavailability profile based on their lipophilicity, size, polarity, solubility, flexibility, and saturation properties. Because ABT239 is the compound with the fewest violations of the different drug-likeness rules, followed by bortezomib with only one violation, these two drugs stand out from the others due to their good ADME profile. Finally, after observing the toxicity predictions for CYP-inhibition enzymes, bortezomib stands out as the only compound that does not inhibit any of the cytochromes studied. Taking all this into account, considering that it is not necessary for the drug to penetrate the BBB, bortezomib, which had already stood out with a selectivity index 100 times higher than the reference drug miltefosine, stands out as the best of the candidates in this work.

## 4. Materials and Methods

### 4.1. Chemicals

The molecules, stored at −20 °C, were supplied diluted in DMSO at 10 mM. Detailed data concerning the Pathogen Box compounds can be found at https://www.mmv.org/pathogen-box (accessed on 30 May 2021).

After an initial screening, five pure compounds were purchased: Terconazole was obtained from Cymit Química S.L. (Pamplona, Spain); ABT-239 was purchased from MedChemExpress (Monmouth Junction, NJ, USA); and Bortezomib, Almitrine mesylate, and Midostaurin hydrate were purchased from Sigma-Aldrich (now Merck) (Darmstadt, Germany).

### 4.2. Strains

For the experiments, *Leishmania amazonensis* strain (MHOM/BR/77/LTB0016) and *Leishmania donovani* (MHOM/IN/90/GE1F8R) promastigotes were cultured in Schneider’s medium (Sigma-Aldrich, Darmstadt, Germany) supplemented with 10% fetal bovine serum at 26 °C and grown to the log phase. Parasites were also cultured in RPMI 1640 medium (Gibco, Waltham, MA, USA) at 26 °C. Murine macrophages J774A.1 (ATCC #TIB-67) were maintained in DMEM medium at 37 °C with 5% CO_2_ and used for cytotoxic activity assays.

### 4.3. Leishmanicidal Activity

Initial screening of the 160 compounds against *Leishmania* promastigotes was performed at 10 μM following MMV foundation instructions provided with the box. The compound activity was assessed using the modified alamarBlue^®^ assay (Invitrogen/Life Technologies, Madrid, Spain) with 10^6^ promastigotes per well. DMSO concentration never surpassed 0.1% (*v*/*v*) to avoid impacting parasite proliferation or morphology. Subsequently, an EnSpire multimode plate reader (PerkinElmer, MA, USA) was used to measure fluorescence after 72 h. Compounds exhibiting over 90% inhibition were further analyzed for their IC_50_. Serial dilutions were made in 96-well plates and incubated with parasites for 72 h after adding alamarBlue^®^. IC_50_ values were calculated using SigmaPlot 14.0 (Systat Software Inc., Chicago, IL, USA) based on fluorescence measurements [27]. Each concentration was tested in duplicate across three independent experiments.

Intracellular amastigote activity against *L. amazonensis* was measured through a parasite rescue and transformation assay for the purchased compounds. Macrophages were seeded in a 96-well plate and infected with promastigotes at the metacyclic stage for subsequent compound treatment. After incubation, macrophages were lysed, and the medium and conditions were changed to stimulate amastigote transformation. The results were assessed using alamarBlue^®^ [28].

### 4.4. Cytotoxic Activity

Cytotoxicity of the selected molecules was evaluated following a protocol similar to the leishmanicidal activity assessment. Murine macrophages were used in this assay. After incubation with the compounds for 24 h, alamarBlue^®^ was added, and CC_50_ values were calculated [29].

### 4.5. Variations in ATP Levels

Cellular ATP level alterations due to treatments were detected using CellTiter-Glo (Promega). Promastigotes were incubated with compound IC_90_ for 24 h and then analyzed for luminescence using the EnSpire spectrophotometer [30]. This assay was carried out in three separate and independent experiments.

### 4.6. Mitochondrial Membrane Potential Disruption

JC-1 Mitochondrial Membrane Potential kit (Cayman Chemical, Ann Arbor, MI, USA) was employed following the manufacturer’s instructions. Parasites were treated with compound IC_90_ for 24 h and then assessed using JC-1 dye [31]. Three independent experiments were conducted for this assay.

### 4.7. Reactive Oxygen Species (ROS) Detection

ROS accumulation was determined using CellRox DeepRed (Invitrogen) fluorescent dye. Parasites were incubated with compound IC_90_ for 24 h, followed by CellRox staining and observation under a fluorescent microscope. This staining was performed three times in independent tests, with a positive control using hydrogen peroxide to induce ROS production [32].

### 4.8. Plasmatic Membrane Permeability

To assess changes in membrane permeability, the SYTOX^®^ Green assay was conducted on the parasites. Initially, promastigotes were exposed to the compounds’ IC_90_ and incubated at 26 °C for 24 h. SYTOX^®^ Green was introduced at a final concentration of 1 μM (Molecular Probes^®^, Thermo Fisher Scientific, Waltham, MA, USA) for 30 min in darkness. Following this, the protozoa were transferred to black plates, and fluorescence was quantified using an EnSpire^®^ Multimode Plate Reader (Perkin Elmer, Madrid, Spain) at an excitation wavelength of 504 nm and emission wavelength of 523 nm. To ensure reliability, this experiment was repeated at least three times on separate days. The rise in fluorescence correlated with the binding of the dye to the promastigote’s DNA. Positive control was executed as the internal benchmark, achieved by completely permeabilizing the cells with the addition of 0.1% Triton X-100 [33].

### 4.9. Chromatin Condensation Evaluation

In the assessment of chromatin condensation in treated parasites, we utilized the Invitrogen Chromatin Condensation/Dead Cell Apoptosis Kit, incorporating Hoechst 33,342 and Propidium Iodide (PI). The parasites were exposed to a 30-min incubation at a temperature of 26 °C in complete darkness following the introduction of the kit. The outcomes were observed using the EVOS fluorescence microscope, employing DAPI with an excitation wavelength of 350 nm and an emission wavelength of 461 nm for Hoechst. Additionally, RFP was used with an excitation wavelength of 535 nm and an emission wavelength of 617 nm for PI. The acquired images were classified into three distinct results: minimal fluorescence for both stains indicating viable cells, intense blue fluorescence suggesting chromatin condensation (programmed cell death), and vibrant red fluorescence signifying cell demise [34].

### 4.10. ADME-Tox Predictions

To compute physicochemical descriptors as well as to predict in silico ADME and toxicity parameters, pharmacokinetic properties, drug-like nature, and medicinal chemistry friendliness of one or multiple small molecules to support drug discovery, SwissADME (developed and maintained by the Molecular Modeling Group of the Swiss Institute of Bioinformatics) web tool was utilized [35].

### 4.11. Statistical Analyses

The data are displayed as mean ± standard deviation (SD) derived from a minimum of three independent experiments, representing the outcomes consistently obtained. Inhibitory concentrations (IC_50_ and CC_50_) were computed via non-linear regression analysis, accompanied by 95% confidence limits. Statistical variance amidst means was assessed employing a one-way analysis of variance (ANOVA) for three or more samples, followed by Tukey’s test for pairwise comparisons of means facilitated by SigmaPlot 12.0 software. A significance level of *p* < 0.05 was considered for all analyses.

## 5. Conclusions

In response to the acute need for innovative therapies for leishmaniasis, this research aimed to identify compounds from the COVID Box with potential efficacy against two *Leishmania* species, resulting in the discovery of four promising molecules: bortezomib, almitrine, terconazole, and ABT239. These compounds demonstrated notable inhibitory effects, exhibiting selectivity, particularly bortezomib, more than 100 times greater than the reference drug miltefosine. Mechanistic studies revealed their ability to induce programmed cell death in *Leishmania* parasites, minimizing unregulated cell death and inflammatory responses. The compounds also displayed favorable pharmacokinetic and drug-likeness profiles, with bortezomib emerging as the safest candidate based on toxicity predictions. Beyond leishmaniasis, their diverse pharmacological properties suggest potential efficacy against other pathogenic microorganisms, highlighting the importance of repurposing existing drugs for neglected tropical diseases. These findings emphasize the potential of the COVID-Box in drug discovery and the importance of collaborative efforts in addressing global health challenges.

## Figures and Tables

**Table 1:** Leishmanicidal activity against the promastigote stage of the selected compounds from MMV COVID Box. Values correspond to inhibitory concentrations 50 ± standard deviation (μM). w/a: without activity.

Compounds	*L. amazonensis*	*L. donovani*
Bortezomib	0.42 ± 0.08	37.20 ± 2.51
Almitrine	1.19 ± 0.02	w/a at 100
Terconazole	3.82 ± 1.04	11.61 ± 1.38
Midostaurin	0.67 ± 0.05	w/a at 100
ABT239	1.31 ± 0.07	1.68 ± 0.25
Miltefosine	6.48 ± 0.24	72.19 ± 3.06

**Table 2:** 2D Chemical structures of the selected active compounds.

Bortezomib	Almitrine
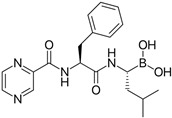	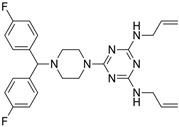
Terconazole	Midostaurin
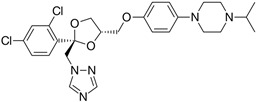	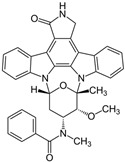
ABT239	
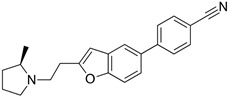	

**Table 3:** Leishmanicidal activity against the amastigote stage of the selected compounds from MMV COVID Box and cytotoxic activity against murine macrophages (μM). Selectivity index (CC_50_/IC_50_). w/a: without activity; n/d: not determined.

Compounds	*L. amazonensis*	Murine Macrophages	Selectivity Index
Bortezomib	0.04 ± 0.01	>100	2500
Almitrine	2.18 ± 0.47	>100	46
Terconazole	2.38 ± 0.40	45.01 ± 8.27	19
ABT239	0.49 ± 0.13	22.63 ± 4.38	46
Miltefosine	3.12 ± 0.30	72.19 ± 3.06	23

## Data Availability

Data is contained within the article and Supplementary Material.

## Supplementary Materials

The following supporting information can be downloaded at https://www.mdpi.com/article/10.3390/ph17030266/s1, Figure S1: Bioavailability Radar appraisal of drug-likeness of the selected molecules. The pink area represents the optimal range for each properties (lipophilicity: XLOGP3 between −0.7 and +5.0, size: MW between 150 and 500 g/mol, polarity: TPSA between 20 and 130 Å^2^, solubility: log S not higher than 6, saturation: fraction of carbons in the sp3 hybridization not less than 0.25, and flexibility: no more than 9 rotatable bonds.
