# Supplementary material for: Anti-COVID Drugs (MMV COVID Box) as Leishmanicidal Agents: Unveiling New Therapeutic Horizons
Source: Pharmaceuticals (Basel). 2024 Feb 20;17(3):266. doi: 10.3390/ph17030266 (PMC10974317; doi:10.3390/ph17030266)
Supplement: Supplementary file 1 [file pharmaceuticals-17-00266-s001.zip › pharmaceuticals-2862852-supplementary.pdf]

Supplementary Information

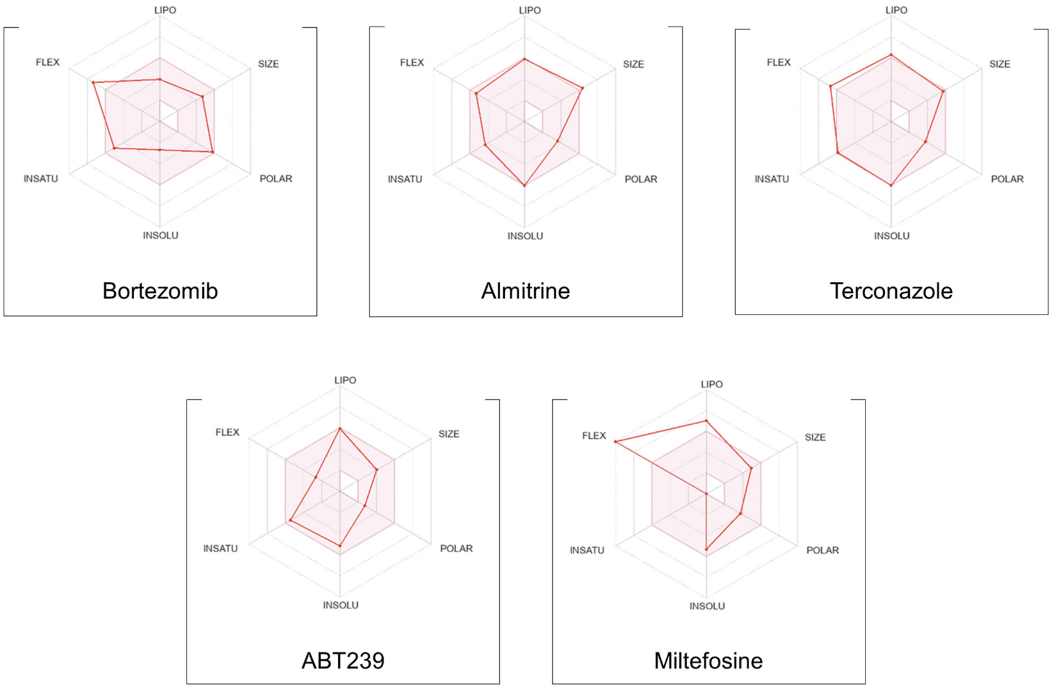

Supplementary Figure S1. Bioavailability Radar appraisal of drug-likeness of the selected molecules. The pink area represents the optimal range for each properties (lipophilicity: XLOGP3 between −0.7 and +5.0, size: MW between 150 and 500 g/mol, polarity: TPSA between 20 and 130 Å2, solubility: log S not higher than 6, saturation: fraction of carbons in the sp3 hybridization not less than 0.25, and flexibility: no more than 9 rotatable bonds.
